# Ascites in the Puerperium in the Context of a Woman with Turner Syndrome Who Conceived through Assisted Reproductive Technology

**DOI:** 10.1155/2015/459679

**Published:** 2015-10-22

**Authors:** Nikolaos Tsagkas, George Valasoulis, Konstantinos Zikopoulos, Calliope Zerzi, Ioannis Mitselos, Ioannis Koutoulakis, Nikolaos Tzampouras, Theodor Stefos

**Affiliations:** ^1^Department of Obstetrics & Gynaecology, University Hospital of Ioannina, Stavros Niarchos Avenue, 45500 Ioannina, Greece; ^2^Department of Gastroenterology, University Hospital of Ioannina, Stavros Niarchos Avenue, 45500 Ioannina, Greece

## Abstract

The case is about a young female who delivered twins by caesarean section (CS). On the 4th postoperative day, she presented with ascites which was resistant to empirical antibiotic and diuretic treatment. The woman was affected by Turner syndrome (TS); she had a medical background of chronic use of hormonal medication since puberty and conceived through ART- (assisted reproduction techniques-) IVF-oocyte donation. It is important to exhibit high suspicion for clot formation in the hepatic vasculature during the puerperium, especially in the case of history of chronic hormone treatment. Ascites albumin gradient and Doppler values lead to the diagnosis of thrombosis and the administration of high doses of anticoagulants is considered to be fundamental.

## 1. Case Presentation

A 26-year-old multipara woman (1.51 m height, 70 kg weight, and high BMI = 30.7), G2P4, was admitted after delivering twins by CS at 36^+2^/40 weeks of gestation.

**Figure 1:**
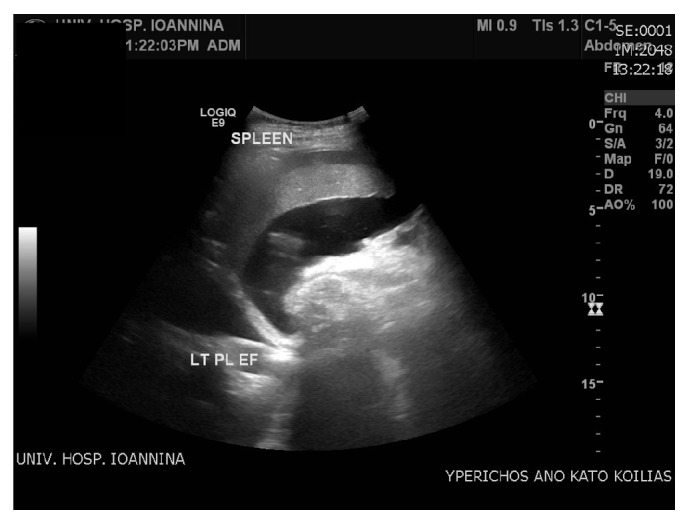
Ascites (fluid accumulation in the peritoneal cavity).

After consultation, a decision was taken for elective CS due to twin pregnancy and the history of previous CS. No complication was apparent at the time of the procedure; however, on the 4th postoperative day, the patient presented with high temperature (38.8° Celsius). Blood cultures were obtained and empirical intravenous antibiotic treatment was initiated. Since the patient had at least 5 risk factors for venous thromboembolism (VTE) in pregnancy and puerperium (high BMI >30 based on booking weight, multiple pregnancy, assisted reproductive therapy (ART), elective CS, and preterm delivery), she was on prophylactic dose of low molecular weight heparin (LMWH) which was further extended postpartum as per British RCOG Green-top Guideline No. 37a. She also complained of bloating and upper right quadrant abdominal pain. At the physical examination her legs were swollen and there was a suspicion of peritoneal fluid collection. This was confirmed by ultrasound, as the sonographic features were suggesting ascites rather than bleeding within the peritoneal cavity (Figure 1). Besides that, haemoglobin in consecutive blood samples was retained within normal limits and without fluctuation. Bleeding as a postoperative complication was excluded at that point. Possible injury of the ureters or the urinary bladder at the time of CS was excluded by Computer Tomography urography (CT-urography), and the Magnetic Resonance Imaging (MRI) of the upper and lower abdomen confirmed the ascites and revealed a moderate splenomegaly (largest spleen dimension 16 cm).

Ascites drainage was attempted unsuccessfully transabdominally. The transvaginal approach that followed proved to be adequate and 2.5 liters of macroscopically clear or rather yellowish ascetic fluid was gradually drained. A sample was sent for pathology investigation and biochemical workout (Gram stain, cultures, tuberculosis testing, and cytological and biochemical analysis). At the same time, a blood sample was sent for biochemical analysis in order to define the serum-ascites albumin gradient.

**Figure 2:**
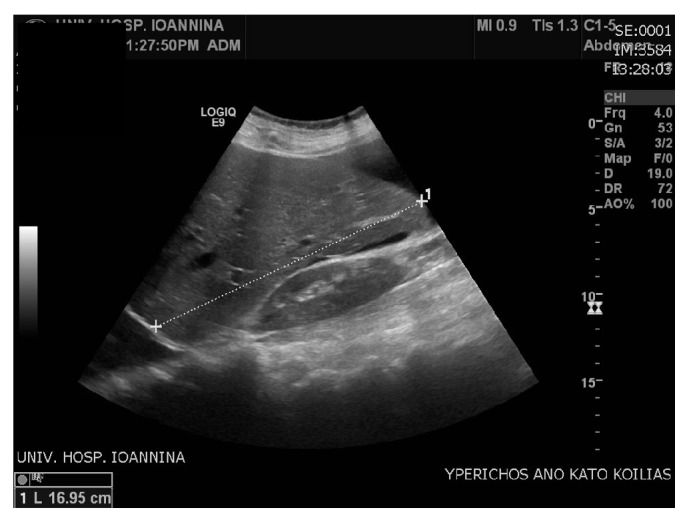
Hepatomegaly.

**Figure 3:**
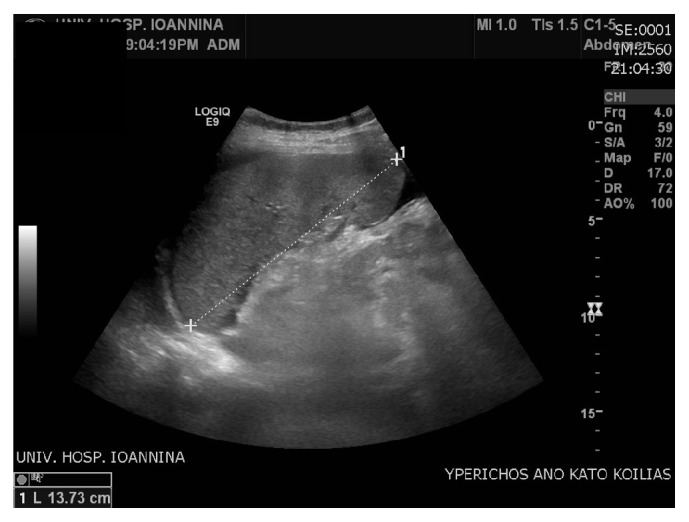
Splenomegaly.

**Figure 4:**
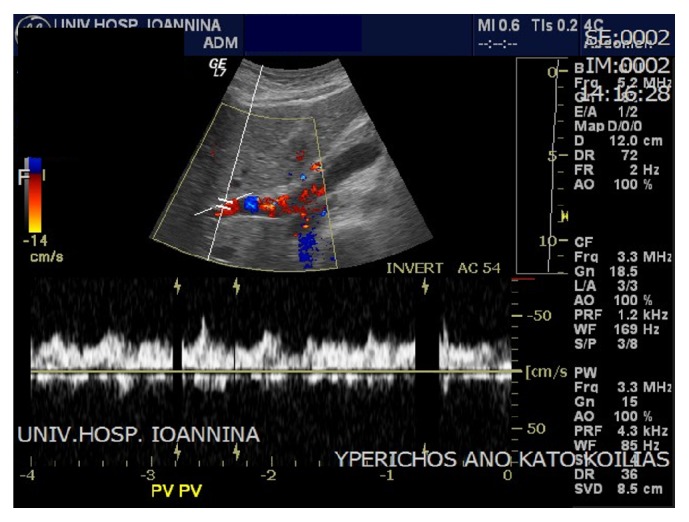
Reversed flow in the portal vein.

The results came back with albumin gradient levels 1.6 g/dL (>1.1 g/dL) suggesting portal hypertension. Draining of the ascites led to relief of the symptoms, but few days later ascites recurred. The most likely scenario of a “recurring persisting ascites post CS,” after excluding the obvious surgical causes, is to be thrombosis of the hepatic veins. This was consistent with the patient's history, that is, the findings of hepatosplenomegaly (Figures 2 and 3) and the abnormal Doppler values (Figure 4), where abnormal hepatic blood vessels flow waveform was showed.

Regarding the Doppler findings, the portal vein slow flow (velocity < 16 cm/sec) as well as the reversed flow characterizes portal hypertension [1] (Figure 4). This portal vein flow can also be described as “hepatofugal” or “retrograde.” This reverse flow occurs when the normal pressure within the vein is distorted and the backpressure exceeds the forward pressure. In Figure 4 the waveform of the portal vein is shown to be below the baseline and this is a diagnostic feature for portal hypertension [2, 3].

## 2. Summary

A case of a Turner (XO) syndrome woman is presented. There was no deep venous thromboembolism (DVT) event disclosed either in the family or in the personal history but a personal history of chronic hormonal treatment was noted. She had been receiving hormonal treatment with combined estrogen/progesterone medication (estradiol valerate + norgestrel and drospirenone + ethinylestradiol) in order to achieve cyclical monthly menstruation, since the age of 15. Three years ago, she had a twin pregnancy by IVF (oocytes donation), with no complications antenatally, delivered by elective CS, and no complications were observed in the postnatal period. A second twin pregnancy followed again by IVF. This also came to term uneventfully and the woman delivered a second set of twins.

**Figure 5:**
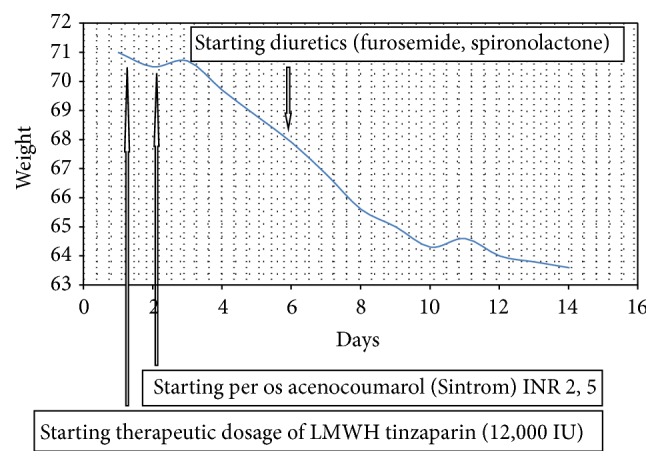
Weight decrease resembling ascites remission.

On the 4th postoperative day she developed fever, upper abdominal pain, and ascites; she received intravenous course of antibiotics (cefuroxime, metronidazole) and was on prophylactic dose of LMWH (tinzaparin, 3,500 IU). The personal history of chronic hormonal treatment and the persistent ascites in the postpartum period after CS in addition to the portal hypertension index (serum-ascites albumin gradient) and the abnormal waveform of the portal vein blood flow on the Doppler findings lead to the diagnosis of hepatic vasculature thrombosis. Following that, a therapeutic dose for DVT was initiated (tinzaparin 12,000 IU) and ascites started to reduce as shown by the ultrasound findings and the daily decrease of body weight. Two days later, warfarin treatment was initiated and five days later, an additional combined treatment with diuretics (furosemide and spironolactone) was started, enhancing ascites remission (Figure 5).

The overall condition of the patient was improved and she was discharged afebrile and free of any symptoms. In the discharge summary, a clear follow-up plan was made for repeat hepatosplenic triplex in six months' time. In parallel, levothyroxine medication for hypothyroidism was prescribed as well as continuous warfarin treatment (aiming in INR 2.5 to 3) for at least 6 months' period. Antidiuretics were suggested to be continued (furosemide and spironolactone) and a clear recommendation to avoid any hormonal regimen (estrogen/progesterone) was given. Thrombophilia screening was advised to be repeated at a later date because the protein C, S values are not reliable in pregnancy. In addition, proteins C, S may be low when treatment with warfarin or heparin is administered or may not be reliable during the acute phase of thrombosis [4, 5].

## 3. Discussion

The combination of several parameters (delivery by caesarean section, pregnancy per se/puerperium, chronic hormone treatment for initiation and maintenance of menstruation (hypergonadotropic hypogonadism), IVF, and twin pregnancy in the context of Turner syndrome) can lead to DVT. In detail, it has been documented that DVT is the leading cause of maternal mortality, caesarean section is a well-known risk factor, and the puerperium is well established as the period in which the highest suspicion has to be exhibited [6].

## 4. Hormone Treatment

Only 5–25% of women with Turner syndrome experience thelarche and/or adrenarche without any medical treatment and just 2–5% may undergo menarche. On the other hand, natural conception and pregnancy occur only in 2–10% of the cases [7]. Because of the ovarian insufficiency, estrogen therapy should be given in order to achieve feminization, development of secondary female sex characteristics, and uterine development. In our case, hormone supplementation started at the age of 15 in the form of combined oral contraceptive medication which can both act as HRT and establish cyclic monthly menstruation [8, 9].

Apart from the reproductive side, administration of HRT is also important for the prevention of osteoporosis and cardiovascular wellbeing in later life. However, it also poses some risks. Evidence suggests a 2- to 3-fold increase when HRT is used and this risk may be even higher specifically with combined estrogen/progesterone medication. Thus, in our case, chronic hormone treatment constitutes a risk factor for thrombosis [10].

## 5. IVF

As it has already been mentioned, the vast majority of women with Turner syndrome are destined to face premature ovarian failure sooner or later. Regarding fertility in that population, the available options are either IVF-follicular stimulation and oocyte retrieval before the diminishing of ovarian reservoir or IVF-oocyte donation cycle. Recent studies have underlined an increased incidence of venous thromboembolism in IVF pregnancies. Of special note is that the risk is higher: firstly antenatally in the 1st trimester and secondly postnatally, up to 6 weeks after delivery.

## 6. Twin Pregnancy

Pregnancies achieved by assisted reproduction methods pose a higher risk of thrombosis and the same applies in multiple pregnancies which also pose a higher risk of thrombosis (when compared to singleton pregnancies) [11]. Because of that, it is highly recommended in IVF cycles to proceed to single embryo transfer, and this particularly applies in TS. Specifically women with TS should follow a single embryo transfer plan because they compose a high risk pregnancy group, as they are of small stature with a small pelvis (premature delivery) and they also have increased risk of thrombosis due to concurrent pathologies, such as congenital cardiovascular (aortic) abnormalities [12].

## 7. Turner Syndrome: Thrombosis Risk

As for the Turner syndrome per se, it is not generally considered as a condition that predisposes to thrombosis but there are data in the literature, which show low protein C values, low protein S values, and high fibrinogen concentration. Deficiencies of proteins C, S are widely recognized as (hereditary/acquired) disorders which increase the risk of venous thrombosis. Protein C deficiency has been documented in about 0.5–0.2% in the general population and protein S in 0.2% [5]. In a study of 82 Turner syndrome individuals, 3.7% had protein C deficiency and 14.81% protein S deficiency and these percentages are higher than in the general population. The authors conclude that before starting HRT in TS it would be good medical practice to perform thrombophilia screening [13].

Furthermore, when the levels of factor VIII of the coagulation cascade are above 150 IU/dL, there is a 4 times higher risk of thrombosis [14]. Related to that, in a case report of a woman with TS who presented with portal vein thrombosis it has been shown that factor VIII was elevated, as well as the v-Willebrand factor [15]. This has also been reported in a second case report of a 14-year-old girl from Libya who presented with portal vein thrombosis and the factors VIII and v-Willebrand were found to be elevated [16]. Further investigations in TS cases without thrombotic incidents documented that the TS subpopulation of non-O blood group exhibit higher factor VIII values than a control group of normal women [15].

Finally, there is also a TS subgroup who presents with unexplained liver test abnormalities. The hepatic biopsies have shown that there is a histologically defined vascular disorder of the liver and liver malfunction can lead to thrombosis [8]. In conclusion, Turner syndrome may be a condition of high thrombotic risk as the above-mentioned studies imply and this can be a reason which leads in our case to hepatic vein thrombosis and persistent ascites.
